# Effects of the flow diversion technique on nucleotide levels in intra-cranial aneurysms: A feasibility study providing new research perspectives

**DOI:** 10.3389/fcvm.2022.885426

**Published:** 2022-09-14

**Authors:** Omer F. Eker, Boris Lubicz, Melissa Cortese, Cedric Delporte, Moncef Berhouma, Bastien Chopard, Vincent Costalat, Alain Bonafé, Catherine Alix-Panabières, Pierre Van Anwterpen, Karim Zouaoui Boudjeltia

**Affiliations:** ^1^Department of Neuroradiology, Hôpital Pierre Wertheimer, Hospices Civils de Lyon, Lyon, France; ^2^CREATIS Laboratory, UMR 5220, U1206, Université Lyon, INSA-Lyon, Université Claude Bernard Lyon 1, UJM-Saint Etienne, CNRS, Inserm, Lyon, France; ^3^Department of Interventional Neuroradiology, Erasme Hospital, Université Libre de Bruxelles, Brussels, Belgium; ^4^RD3–Pharmacognosy, Bioanalysis, and Drug Discovery and Analytical Platform, Faculty of Pharmacy, Université Libre de Bruxelles, Brussels, Belgium; ^5^Department of Vascular Neurosurgery, Hôpital Pierre Wertheimer, Hospices Civils de Lyon, Lyon, France; ^6^Scientific and Parallel Computing Group, CUI, University of Geneva, Geneva, Switzerland; ^7^Department of Neuroradiology, Hôpital Gui de Chauliac, Montpellier, France; ^8^Laboratory of Rare Human Circulating Cells, University Medical Center of Montpellier, University of Montpellier, Montpellier, France; ^9^CREEC, MIVEGEC, University of Montpellier, CNRS, IRD, Montpellier, France; ^10^Laboratory of Experimental Medicine (ULB 222), Medicine Faculty, Université Libre de Bruxelles, CHU de Charleroi, Charleroi, Belgium

**Keywords:** intra-cranial aneurysm, flow diversion, nucleotides, *in situ* blood collection, thrombosis – etiology

## Abstract

**Introduction:**

The flow diverter stent (FDS) has become a first-line treatment for numerous intra-cranial aneurysms (IAs) by promoting aneurysm thrombosis. However, the biological phenomena underlying its efficacy remain unknown. We proposed a method to collect *in situ* blood samples to explore the flow diversion effect within the aneurysm sac. In this feasibility study, we assessed the plasma levels of nucleotides within the aneurysm sac before and after flow diversion treatment.

**Materials and methods:**

In total, 14 patients with unruptured IAs who were selected for FDS implantation were prospectively recruited from February 2015 to November 2015. Two catheters dedicated to (1) FDS deployment and (2) the aneurysm sac were used to collect blood samples within the parent artery (P1) and the aneurysm sac before (P2) and after (P3) flow diversion treatment. The plasma levels of adenosine monophosphate (AMP), adenosine diphosphate (ADP), and adenosine triphosphate (ATP) at each collection point were quantified with liquid chromatography and tandem mass spectrometry.

**Results:**

The aneurysms were extradural in nine (64.3%) patients and intra-dural in five (35.7%) patients. They presented an average diameter of 15.5 ± 7.1 mm, height of 15.8 ± 4.6 mm, and volume of 2,549 ± 2,794 ml. In all patients (100%), 16 FDS implantations and 42 *in situ* blood collections were performed successfully without any complications associated with the procedure. The ATP, ADP, and AMP concentrations within the aneurysm sac were decreased after flow diversion (*p* = 0.005, *p* = 0.03, and *p* = 0.12, respectively). Only the ATP levels within the aneurysm sac after flow diversion were significantly correlated with aneurysm volume (adjusted *R*^2^ = 0.43; *p* = 0.01).

**Conclusion:**

*In situ* blood collection within unruptured IAs during a flow diversion procedure is feasible and safe. Our results suggest that the flow diversion technique is associated with changes in the nucleotide plasma levels within the aneurysm sac.

## Introduction

The flow diversion technique is recognized as a safe and efficacious first-line therapy for selected intra-cranial aneurysms (IAs). Although initially dedicated to the treatment of large or giant complex IAs in proximal intra-cranial arteries (i.e., the internal carotid or vertebral arteries), this technique has been expanded to various types of aneurysms and locations, such as ruptured aneurysms (1). Unlike surgical clipping and endovascular coiling, which target the aneurysm itself, the flow diversion technique relies on the primary endoluminal reconstruction of the parent vessel through the deployment of a flow diverter stent (FDS), thus leading to the secondary occlusion of the aneurysm (1, 2). The aneurysm cure results from intra-saccular thrombosis are favored by this technique and therefore are not immediate but progressive. Indeed, a recent meta-analysis has reported complete occlusion rates of 68% (65–72%) and 90% (88–92%) with this technique in follow-up before 6 months and at 6–12 months, respectively (3, 4).

Despite the increasing use of FDSs in recent years and the introduction of newer-generation surface-modified FDSs, the mechanism of flow diversion and its therapeutic effects remain unclear. Two mechanisms are commonly understood to be involved in FDS action: (1) the hemodynamic alteration in the aneurysm sac induced by the flow redirection within the parent vessel and (2) the promotion of endothelialization at the aneurysm neck favored by the implant acting as a “scaffold” that increases endothelial cell migration and colonization (2, 5, 6). These two mechanisms, dependently or independently, have been proposed to explain the intra-saccular thrombosis resulting from the treatment and leading to IA cure. Numerous studies exploring these two mechanisms have improved the understanding of this technique (2, 7–9). However, they have not provided a comprehensive picture of the flow diversion effect that may explain why as many as 10% of IAs treated with FDSs remain patent at 1-year follow-up (4).

Little is known regarding the biological phenomena induced by the flow diversion technique within the IA. Notably, the effect of intra-saccular blood stasis on platelet aggregation remains unknown. Nucleotides (intra- and/or extracellular) play diverse physiological roles but are pathological under certain conditions (10). The role of adenosine diphosphate (ADP) in platelet aggregation through the P2Y12 receptors is well known, and many antiplatelet therapies target its action (11). Adenosine triphosphate (ATP) is released from erythrocytes and platelets under certain pathophysiological conditions, such as hypoxia or venous stasis (12). ATP is also known to induce platelet aggregation in whole blood *via* conversion to ADP by ecto-ATPases on leukocytes (11).

In this article, we propose an original investigative technique to collect blood samples from the aneurysm sac during endovascular treatment (EVT) of unruptured IAs with the flow diversion technique. We demonstrated its feasibility in patients treated with FDSs for unruptured IAs. The collected blood samples were analyzed to assess the levels of intra-saccular nucleotides before and after flow diversion treatment.

## Materials and methods

### Population

In total, 14 patients with unruptured IAs who were selected for FDS implantation were prospectively recruited from February 2015 to November 2015 in two INR centers. The indications for FDS implantation were assessed after a multidisciplinary meeting at the relevant institution for all patients. Local ethics committee guidelines were followed for this study (DGRI CCTIRS MG/CP 2012.528; Comité d’Ethique du CHU de Lyon; Lyon/France). Informed consent was obtained from all patients. This work was funded partly by the THROMBUS VPH Project (7th Framework Programme/Seventh Framework Programme of European Commission/Virtual Physiological Human ICT-2009.5.3/Project reference: 269966; http://www.thrombus-vph.eu).

### Aneurysm treatment

All patients were treated under general anesthesia with a biplane angiographic system (Phillips Allura, Philips, Best, Netherlands) after preparation according to the institutional protocol common to both centers (loading dose of 300 mg of clopidogrel administered 1 day before EVT; systemic heparinization administered during the endovascular procedure and stopped at the end of the treatment; per-procedural loading dose of 300 mg of acetylsalicylic acid after FDS deployment; and double antiplatelet therapy initiated for 6 months starting on day 1 after treatment, with 75 mg of acetylsalicylic acid and 75 mg of clopidogrel per day). The aneurysm and parent vessel underwent 3D rotational angiography before the EVT, thus allowing for 3D reconstruction and treatment planning. One or more FDSs were deployed in one session according to the aneurysm neck size (Pipeline™ Embolization Device, PED™, ev3-Covidien, Irvine, CA, United States; Flow Redirection Endoluminal Device, FRED™, Microvention Terumo, Aliso Viejo, CA, United States; and p64 Flow Modulation Device, Phenox, Germany). If deemed necessary by the interventional neuroradiologist, additional coiling was performed (Target Coils, Stryker Neurovascular, Fremont, CA, United States).

### Aneurysm assessment

The 3D aneurysms and parent vessel geometries were segmented and reconstructed from the 3D angiographic acquisition before the EVT (spatial resolution 0.48 mm × 0.48 mm), according to a new active contour method dedicated to the near real-time segmentation of 3D objects with the level-set method (13). This method allowed for the calculation of the two maximal diameters (mm), the depth (mm), the neck size (mm), and the volume (i.e., the volume of the patent intra-saccular lumen; mm^3^) of all aneurysms in dedicated software (ITK-SNAP, Penn Image Computed and Science Laboratory, University of Pennsylvania, United States).

### *In vivo* intra-aneurysmal blood collection

The principle of the technique relies on using the catheter normally dedicated to the FDS deployment and to coiling for the blood collection. During the EVT, a 0.027-inch Marksman Microcatheter (ev3 Neurovascular, Irvine, CA, United States) dedicated to FDS deployment was positioned within the parent artery and allowed for blood collection at the P1 position (i.e., parent artery catheter, PAC). The catheter was positioned upstream of the target IA for blood collection. The PAC was then positioned in the parent artery downstream of the IA for FDS deployment. Before FDS deployment, a 0.021-inch Headway Microcatheter (Microvention Terumo, Aliso Viejo, CA, United States) normally dedicated to coiling was positioned within the aneurysm sac and allowed for blood collection at this position (i.e., intra-IA catheter, IIAC). The deployment of the FDS while the IIAC was within the aneurysm lumen enabled the aneurysm neck to be covered and the IIAC to be jailed. The intra-aneurysmal blood samples were collected *via* the IIAC within the aneurysm sac before and after FDS deployment (blood collection P2 and P3, respectively). All microcatheter navigations were performed with 0.014-inch Synchro Guidewires, which were withdrawn before blood collection (Stryker Neurovascular, Fremont, CA, United States). From each catheter at each location (i.e., P1, P2, and P3), before each blood sampling, the catheter (i.e., either PAC or IIAC) was purged with a 1-cc Luer lock syringe (Becton Dickinson, Belgium). The purged volume corresponded approximately to their dead volume space of 0.87 and 0.55 ml for the 0.087-inch and the 0.021-inch catheters, respectively. At the end of the purging, when the blood appeared at the tip of the syringe, a new 1-cc Luer lock syringe was used to collect at least 700 μl of blood. The catheter purging and the blood collection were performed slowly during approximately 30 s of aspiration to minimize the red blood cell (RBC) hemolysis. Thus, three samples per patient were yielded, in the following order, to minimize the intra-luminal device manipulation:

- Within the parent artery upstream of the aneurysm and before the flow diversion (P1);
- Within the aneurysm sac before the flow diversion (P2); and
- Within the aneurysm sac after 10 min of flow diversion (P3).

After collection, the blood samples were collected in 1.5 ml tubes containing citrate and stored at +4°C for less than 2 h. Second, the samples were centrifuged at 3,500 *g* for 10 min, thus allowing for separation and extraction of the serum, which was stored at −80°C until further analyses.

### Biological analyses

In each blood sample, the plasma levels of adenosine monophosphate (AMP), ADP, and ATP were quantified through a liquid chromatography and tandem mass spectrometry method that was previously developed, validated, and fully described by our team (10). This technique provides the advantages of a lower limit of quantification than other methods and the ability to simultaneously quantify all nucleotides within a single injection within less than 10 min on the same blood sample (10).

### Statistical analyses

Categorical variables are expressed as counts and percentages. Continual variables are expressed as mean ± standard deviation (SD). The nucleotide levels at the three blood collection points (P1, P2, and P3) were compared with Student’s *t*-test, the Mann–Whitney rank sum test, the one-way analysis of variance (ANOVA), or the Kruskal–Wallis rank test, according to the results of the Shapiro–Wilk normality test and the Levene test of homogeneity. Linear regression analyses were performed to evaluate any correlation between the nucleotide levels and the aneurysm volume at each blood collection point. A two-sided *p*-value of <0.05 was considered statistically significant. Statistical analyses were performed in R version 3.2 (R Foundation for Statistical Computing, Vienna, Austria) (14).

## Results

**FIGURE 1:**
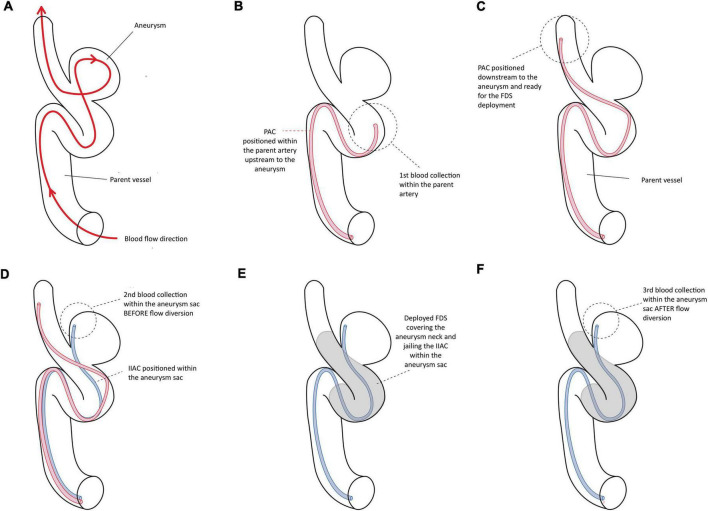
Illustrates the different steps of the blood collection within the parent vessel and the aneurysm sac during the endovascular treatment with the flow diverter stent.

Table 1 summarizes the demographic characteristics of the population, the aneurysm characteristics, the procedural features, and the aneurysm occlusion status at follow-up. Figure 1 shows the blood collection workflow during EVT.

**TABLE 1:** Population and aneurysm characteristics, procedural features, and follow-up.

Case	Age	Sex	Symptoms	Aneurysm characteristics						Procedural characteristics					Occlusion (time)
					
				Localization*	Height (mm)	Diameter 1 (mm)	Diameter 2 (mm)	Volume (mm^3^)	Mural thrombus	n of FDSs	Type of FDS	Coiling	Complications	Contrast media stagnation	
1	30	M	Cavernous sinus syndrome	R ICA C4	12.00	14.00	15.00	1319	No	2	PED™	No	No	Yes	Complete (12 months)
2	55	F	Cavernous sinus syndrome	R ICA C4	24.50	33.50	22.50	9669	No	1	PED™	Yes	No	Yes	Complete (48 months)
3	43	M	Headaches	L ICA C5	20.00	16.00	20.00	3351	No	1	PED™	No	No	Yes	Complete (48 months)
4	79	F	Incidental discovery	L ICA C2	12.00	8.00	9.00	452	No	1	PED™	No	No	Yes	Complete (12 months)
5	50	M	Incidental discovery	L Pericallosal a.	9.00	5.60	7.70	203	Yes	1	PED™	No	No	Yes	Complete (6 months)
6	78	F	Cavernous sinus syndrome	R ICA C4	13.50	12.00	11.50	975	No	1	PED™	No	No	Yes	Complete (12 months)
7	71	F	Cavernous sinus syndrome	R ICA C4	21.00	26.00	23.00	6575	No	1	PED™	No	No	Yes	Complete (48 months)
8	63	F	Cavernous sinus syndrome	L ICA C4	10.00	13.00	18.00	1225	No	1	FRED™	No	No	Yes	Complete (6 months)
9	61	F	Headaches	L ICA C1–C2	18.00	9.50	9.50	851	No	1	PED™	Yes	No	No	Complete (12 months)
10	53	M	Incidental discovery	R ICA C2	18.50	7.80	8.00	604	No	1	PED™	Yes	No	Yes	Complete (12 months)
11	51	F	Incidental discovery	R ICA C3	13.00	19.00	20.00	2587	No	2	P64	No	No	Yes	Complete (6 months)
12	61	F	Headaches	R ICA C2	14.00	7.00	8.00	411	No	1	PED™	Yes	No	No	Complete (12 months)
13	39	M	Cavernous sinus syndrome	R ICA C4	20.00	15.50	14.00	2272	Yes	1	PED™	No	No	Yes	Complete (12 months)
14	58	F	Cavernous sinus syndrome	R ICA C3–C4	16.00	20.00	31.00	5194	Yes	1	PED™	No	No	Yes	Complete (12 months)

F, female; FDS, flow diverter stent; FRED™, flow redirection endoluminal device; ICA, internal carotid artery; L, left; M, male; P64, P64 flow modulation device; PED™, Pipeline™ embolization device; R, right;

*ICA localizations according to Fisher’s classification: C1, communicating segment; C2, ophthalmic segment; C3, clinoidal segment; C4, cavernous segment; C5, intra-petrous.

Table 2 summarizes the nucleotide plasma concentrations at each blood collection point.

**TABLE 2:** Nucleotide plasma concentrations at each blood collection point.

	Blood collection points			*p*-Value
	
	P1	P2	P3	
AMP (μM)	911 ± 520	624 ± 385	600 ± 393	0.12*
ADP (μM)	1,163 ± 286	1,009 ± 283	903 ± 467	0.03*
ATP (μM)	2,566 ± 453	2,158 ± 193	2,049 ± 179	0.005**

P1, parent vessel; P2, intra-aneurysmal before flow diversion stent implantation; P3, intra-aneurysmal after flow diversion stent implantation; ADP, adenosine diphosphate; AMP, adenosine monophosphate; ATP, adenosine triphosphate.

For each measured metabolite, a one-way analysis of variance (ANOVA; *) or the Kruskal–Wallis test (**) was used to compare the mean values among the three blood collection points, according to the results of the Shapiro–Wilk normality test and the Levene test for homogeneity of variance.

**FIGURE 2:**
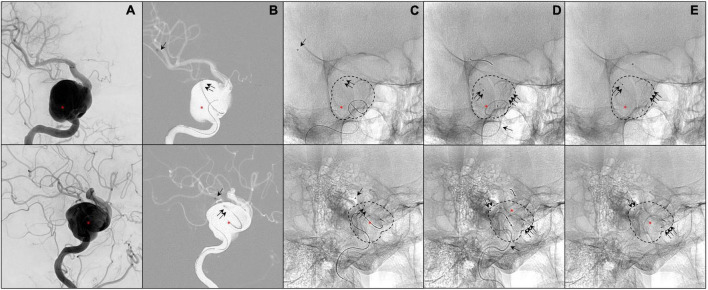
Illustrates a patient presenting a giant aneurysm of the right internal carotid artery (segment C4; red asterisk; **A–E**). The 0.027-inch catheter for the first blood collectio (P1) is visible in panels **(B–E)** (single black arrow). The 0.021-inch catheter within the aneurysm sac is visible in panels **(B–E)** (double black arrows). The flow diverter stent is deployed in panels **(C–E)** (triple black arrows).

Figure 2 illustrates the sampling process in one case (in patient 2).

**FIGURE 3:**
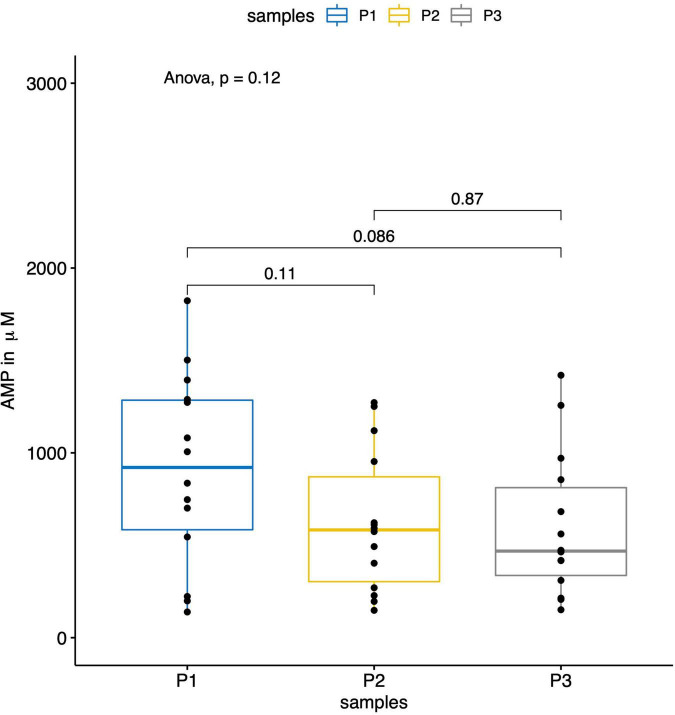
The boxplot shows the results of the measured AMP levels (in μM) within the parent vessels (P1) and the aneurysm sacs before (P2) and after (P3) flow diversion. No significant differences were observed for the AMP levels between the three sampling locations.

**FIGURE 4:**
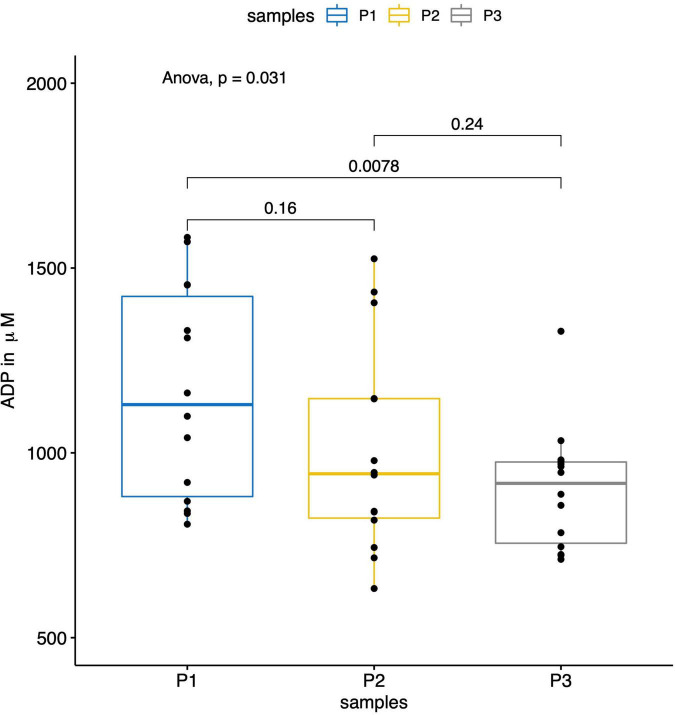
The boxplot shows the results of the measured ADP levels (in μM) within the parent vessel (P1) and the aneurysm sac before (P2) and after (P3) flow diversion. The ADP levels were significantly lower within the aneurysm sacs after flow diversion (P3) compared to the ones in the parent vessels (P1) (*p* = 0.008).

**FIGURE 5:**
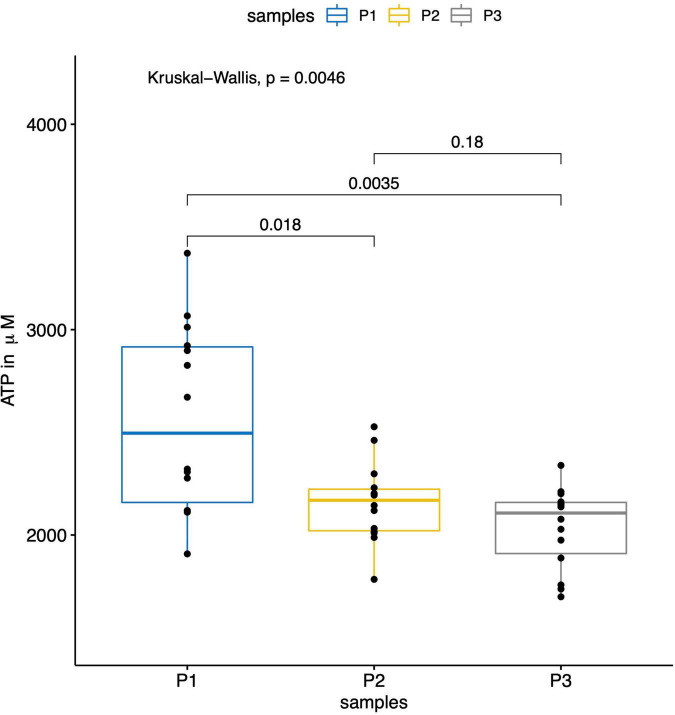
The boxplot shows the results of the measured ATP levels (in μM) within the parent vessel (P1) and the aneurysm sac before (P2) and after (P3) flow diversion. The AMP levels were significantly lower within the aneurysm sacs before (P2) and after (P3) flow diversion compared to the ones in the parent vessels (P1) (*p* = 0.018 between P1 and P2, and *p* = 0.003 between P1 and P3).

Figures 3–5 report the nucleotide levels at each blood collection point.

**FIGURE 6:**
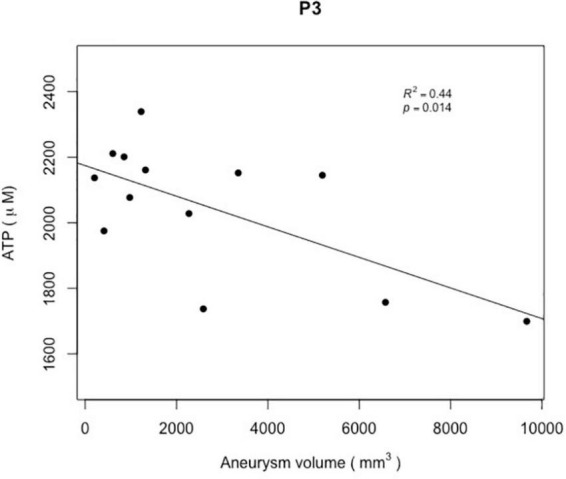
The scatter plot reports the ATP levels (in μM) within the aneurysm sacs after flow diversion (P3) according to the aneurysm volumes (in mm3). A significant correlation was observed between the ATP levels within the aneurysm sacs after flow diversion (P3) and the aneurysm volumes (*R*^2^ = 0.44; *p* = 0.014).

Figure 6 reports the distribution of ATP levels according to the aneurysm volume.

### Study population

In total, 9 (60.0%) patients were women. The median patient age was of 57 ± 15 years (range 30–79 years). Seven (50%) patients had a cavernous sinus syndrome associated with headaches, ipsilateral ptosis, and ophthalmoplegia, due to III, IV, or VI nerve palsy, without any decrease in visual acuity or pupillary abnormalities. Three (21.4%) patients had headaches whose symptoms had no confirmed relationship with their aneurysms. Four (28.6%) patients were asymptomatic, and their aneurysms were incidentally discovered (Table 1). The patients’ medical histories included high blood pressure in three (21.4%) patients, cigarette smoking in six (42.9%) patients, and diabetes mellitus in one (7.1%) patient. No patients presented any vascular steno-occlusive lesions of the supra-aortic trunks or intra-cranial arteries or any hypoxic conditions.

### Aneurysm characteristics

One (7.1%) partially thrombosed aneurysm was located on the left pericallosal artery, and all other aneurysms were located on the right (*n* = 9, 64.3%) and the left (*n* = 4, 28.6%) intra-cranial carotid arteries (Table 1), from their intra-petrous segment to termination. Nine (64.3%) aneurysms were in extradural locations, and five (35.7%) were in intra-dural locations. The maximal aneurysm diameters were 1 and 2, heights and volumes were 14.8 ± 7.8 mm (range 5.6–33.5 mm), 15.5 ± 7.1 mm (range 7.7–31.0 mm), 15.8 ± 4.6 mm (range 9–24.5 mm), and 2,549 ± 2,794 ml (range 203–9,669 ml), respectively. Three (21.4%) aneurysms presented a mural thrombus (Table 1).

### Procedure safety and aneurysm occlusion

In all patients, the intra-arterial and intra-aneurysmal navigations with the IIAC and the PAC were performed successfully. The aneurysms were treated with PED™ in 12 (85.7%), FRED™ in 1 (7.1%), and P64 in 1 (7.1%) cases, respectively. In two (14.3%) patients, two FDSs were deployed in a telescopic fashion to treat the aneurysm. All stents were successfully deployed. Intra-aneurysmal contrast media stagnation after flow direction was observed in 12 (85.7%) patients. In three (21.4%) cases, the aneurysm coil embolization was deemed necessary by the physician and was performed in addition to the flow diversion technique through the jailed IIAC after P3 blood collection (Table 1). Apart from a groin hematoma in one (7.1%) patient, no postoperative complications were observed in all procedures. In all patients (100%), the blood collection through the PAC and IIAC did not affect the EVT and its duration. The aneurysm occlusion was obtained between 6 and 48 months of follow-up in all patients. The occlusion status did not change during the follow-up between 2015 and 2021. No patients presented any clinical consequences of the intra-cranial blood sampling. All patients who initially presented with cavernous sinus syndrome showed clinical improvement or complete regression of their symptoms at the 1-year follow-up. The other patients remained clinically unchanged.

### Biological results

In total, 42 blood collections were successfully performed in 14 patients without any difficulties or per-procedural complications. We observed significantly lower ATP, ADP, and AMP concentrations within the aneurysm sac after flow diversion than within the parent artery and the aneurysm sac before flow diversion (*p* = 0.005, *p* = 0.03, and *p* = 0.01, respectively; Table 2 and Figures 3–5). No differences were observed in the nucleotide levels between smoker (*n* = 6) and non-smoker (*n* = 8) patients. The ATP level within the aneurysm sac after flow diversion was significantly correlated with the aneurysm volume (adjusted *R*^2^ = 0.44, *p* = 0.01; Figure 6 and Supplementary material). No significant correlations were observed between aneurysm volume and ATP levels within the aneurysm sac before flow diversion or within the parent vessel, or AMP and ADP levels at each blood collection point (Supplementary material).

## Discussion

In this work, we used an approach to collect blood samples within the parent artery and the aneurysm sac during EVT for IAs with the flow diversion technique. In this feasibility study, the collected blood was analyzed, and the nucleotide levels were measured. The blood collection had no consequences on the EVT, and there were no complications observed in any patients. First, we observed significant decreases in the ATP and ADP levels within the aneurysm sac after flow diversion. Second, our results showed a significant correlation between the intra-aneurysmal ATP decrease after flow diversion and the aneurysm volume.

The flow diversion technique has revolutionized the treatment of large and complex IAs that were difficult (or even impossible) to treat with previous techniques. Compared with conventional techniques (i.e., coiling, stent-assisted coiling, parent vessel occlusion, or surgical clipping), FDSs showed higher rates of occlusion and lower rates of recurrence without increasing the rate of complications in the treatment of specific aneurysms, such as giant or complex aneurysms (3, 4, 15). Their efficacy relies on the ability to redirect the blood flow out of the aneurysmal sac, thus decreasing the intra-aneurysmal blood flow and the endothelization of the aneurysm neck, hence promoting thrombosis of the aneurysm and its regression (2). Despite the improvements in IA treatment with this technique, its mechanism of action is not fully understood. Previous studies on flow diversion have focused on hemodynamic alterations within the aneurysm sac and/or the endothelization processes within the parent artery that promote IA thrombosis (2, 5, 6). Those studies have not provided information on the intra-saccular biological phenomena occurring after flow diversion. We believe that these phenomena may play a key role in the curative effect of this technique. Better knowledge of the blood modification within the IA induced by flow diversion should aid in understanding its efficacy (or lack thereof) and eventually enable the identification of patients who will not benefit from this technique.

However, any exploration of the blood biology within the IA lumen requires *in situ* real-time blood samples that are not available or accessible in normal conditions or after IA treatment through conventional techniques. A method to obtain sufficient usable blood to explore these mechanisms is lacking. The ideal technique to obtain *in situ* blood samples should meet several criteria. First, it must be safe, posing minimal complication risk to patients. Second, it must be as rapid as possible to prevent or minimize any potential modification of the assessed biological environment by the collection devices or techniques. Third, it must be standardized and reproducible to allow the comparison of blood samples in the same patient or among patients. Fourth, it must be as easy as possible to perform, to enable the dissemination of the technique, and to promote research in this field. For these purposes, we propose a minimally invasive method to achieve this goal while meeting all these criteria. Our approach exploits the flow diversion EVT itself and the materials used during the procedure, i.e., the catheters dedicated to the intra-cranial navigation, the FDS deployment, and additional coiling of the aneurysm if necessary. The catheters are positioned sequentially within the parent artery and the aneurysm sac. The blood collection at each targeted location lasts approximately 30 s through the catheters, a duration compatible with that of EVT. The last collection (i.e., within the aneurysm sac after flow diversion) is performed 10 min after flow diversion, on the basis of previous reports of the changes in nucleotide levels in venous blood after 4 min of stasis (10), to maximize the chances of detecting any changes in nucleotide levels after flow diversion.

The nucleotides in the blood play complex and various roles that are closely associated with local conditions. Indeed, in addition to functioning as an intra-cellular energy source, ATP and ADP are important extracellular signaling molecules (16). Extracellular circulating ATP is rapidly degraded into ADP, AMP, and adenosine by ectonucleotidases (17). ATP and ADP activate P2 receptors on various cells, particularly blood cells, such as platelets and endothelial cells (18, 19), thus regulating several physiological responses. These responses include platelet aggregation, vascular tone (20), and the release of endothelial factors. At least 15 nucleotide-activated cell surface receptors have been found in humans (P2X and P2Y receptors) and show remarkably varied physiological responses.

Platelet aggregation is mediated by ADP through the P2Y12 receptors (21, 22), in a process involving leukocytes, which present cell surface enzymes that degrade ATP into ADP and then AMP, such as the ectonucleoside triphosphate diphosphohydrolase-1 (also known as cluster of differentiation 39, CD39) (23). ATP itself is not considered a platelet-aggregating agent. However, it can induce platelet aggregation when it is added to whole blood (24). In addition, RBCs play a role in platelet aggregation by capturing adenosine (23). These mechanisms may be involved in the progressive intra-aneurysmal thrombosis observed after flow diversion treatment.

Our work showed that ATP and ADP significantly decrease within the aneurysm sac after flow diversion and that aneurysmal volume may influence these phenomena (or at least the ATP decrease). However, given the limited sample size and analyses performed (i.e., measurement of only nucleotide levels), our results cannot explain this observation or indicate a clear conclusion. At most, among the potentially unknown mechanisms triggered by flow diversion, the decreases in ATP and ADP might suggest that flow diversion induces intra-aneurysmal local hypoxia. Indeed, RBCs are known to function as O_2_ sensors, which contribute to the regulation of blood flow and O_2_ delivery. They perform this role by releasing ATP depending on the oxygenation state of hemoglobin and the pH (20, 25–33). In this physiologically important signaling system, when O_2_ decreases, ATP is rapidly degraded to ADP in circulation by ectonucleotidases. The ADP in turn acts on P2Y_13_ receptors on RBCs in a negative feedback pathway for the inhibition of ATP release (34). The increase in ADP levels is also known as a primary mediator of platelet aggregation, thus leading to a sustained response *via* activation of the P2Y12 receptors (21, 22). The rapid degradation of ATP into ADP within the stagnating blood “trapped” outside the FDS might explain the decrease in ATP. After its initial transient increase secondary to the previously described mechanism, the consumption of ADP by the P2Y13 receptors on RBCs and the P2Y12 receptors on platelets might explain the decrease in ADP.

### Limitations

Our work is hypothesis generating but does not provide further answers because of several limitations. First, this was a preliminary feasibility study. Hence, we included only a limited number of patients, and we assessed only the nucleotide levels. The patients had large intra-cranial, mostly internal carotid artery, aneurysms. This design aspect was aimed at minimizing the risk of complications to the patients while maximizing the possibility of successfully collecting blood samples within the aneurysmal sac. Indeed, the internal carotid artery aneurysms are proximal and less prone to accessibility issues than distally located aneurysms. The large aneurysm size also minimized the risk of complications during the intra-aneurysmal catheter manipulation (particularly the risk of aneurysm perforation). Second, we did not evaluate the effects of platelet activation or many other factors with roles in thrombosis, such as the von Willebrand factor, thrombin, thromboxane A2, coagulation activators (such as thrombin-antithrombin complex), and components of the glycocalyx at the endothelial cell surface. Third, we did not consider the dual platelet inhibition required with flow diversion treatment in the analyses of the nucleotide levels. The antiplatelet regimen is commonly based on a combination of acetylsalicylic acid and a P2Y12 inhibitor (i.e., clopidogrel, prasugrel, or ticagrelor) that targets the P2Y12 receptor and therefore may theoretically affect nucleotide levels. For instance, ticagrelor has been reported to induce ATP release from human RBCs (35). Fourth, the intra-aneurysmal flow conditions after flow diversion were also not considered in the analyses of the nucleotide levels. The intra-aneurysmal hemodynamic alterations due to flow diversion markedly vary from no effects (i.e., almost normal patency of the aneurysm) to abrupt stasis. In the first scenario, few or no changes in nucleotide levels within the aneurysm when compared with the parent vessel can reasonably be expected, whereas maximal changes should be expected in stagnating blood. In our work, the intra-aneurysmal contrast media stagnation (indicating blood stagnation) was unevenly distributed and was observed in 85.7% of patients, thus preventing us from drawing any conclusion. Finally, we collected blood within the aneurysm sac after flow diversion at only one time point (i.e., 10 min after the FDS deployment). Sequential and consecutive blood collection might be considered to analyze the kinetics of the intra-aneurysmal biological cascades after flow diversion.

## Conclusion

Blood collection within unruptured IAs during a flow diversion procedure is feasible and appeared safe in our case series. Our preliminary work suggests that flow diversion treatment is associated with changes in plasma nucleotide levels within the aneurysm sac after flow diversion. Further studies in larger populations are needed to better understand the mechanisms involved in thrombus formation after flow diversion.

## Data availability statement

The raw data supporting the conclusions of this article will be made available by the authors, without undue reservation.

## Ethics statement

The studies involving human participants were reviewed and approved by the Comité d’Ethique du CHU de Lyon; Lyon/France. The patients/participants provided their written informed consent to participate in this study.

## Author contributions

OE and KZ contributed equally to the conception and design of the study. OE collected the clinical, biological, and imaging data, performed the imaging post-processing and statistical analysis, and wrote the first draft of the manuscript and the iterative versions. CA-P provided the facilities and means to condition the blood samples after the sampling. All authors contributed substantially to the work described by critically revising the manuscript for important intellectual content.

## Supplementary material

Supplementary Figure 1 The scatter plots report the nucleotide levels (in M) at each location and timepoints (i.e., P1, P2, and P3) according to the aneurysm volume (in mm3). A significant correlation was observed only between the ATP levels within the aneurysm sacs after flow diversion (P3) and the aneurysm volumes (R2 = 0.44; p = 0.014).
