# Supplementary figures and images for: Effects of the flow diversion technique on nucleotide levels in intra-cranial aneurysms: A feasibility study providing new research perspectives
Source: Front Cardiovasc Med. 2022 Sep 14;9:885426. doi: 10.3389/fcvm.2022.885426 (PMC9515454; doi:10.3389/fcvm.2022.885426)

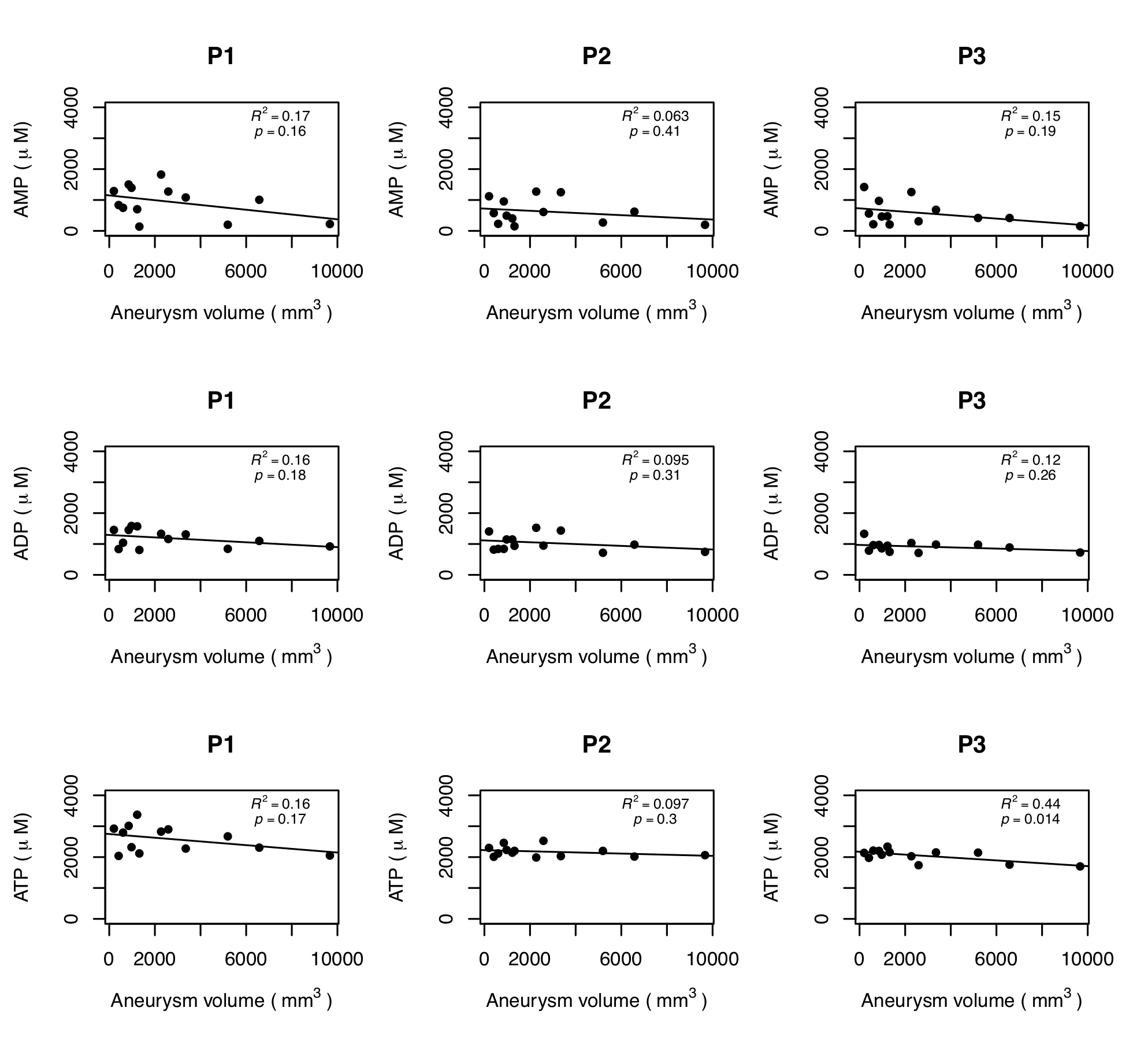

Supplement: Supplementary Figure 1 — The scatter plots report the nucleotide levels (in M) at each location and timepoints (i.e., P1, P2, and P3) according to the aneurysm volume (in mm3). A significant correlation was observed only between the ATP levels within the aneurysm sacs after flow diversion (P3) and the aneurysm volumes (R2 = 0.44; p = 0.014). [file Image_1.JPEG]
